# Sewing needle in the lung

**DOI:** 10.4103/0970-2113.63618

**Published:** 2010

**Authors:** M. Emmanuel Bhaskar, Preetam Arthur

**Affiliations:** *Department of Internal Medicine, Sri Ramachandra Medical College and Research Institute, Porur, Chennai-600 116, India. E-mail: drmeb1974@yahoo.co.in*

**Figure 1:**
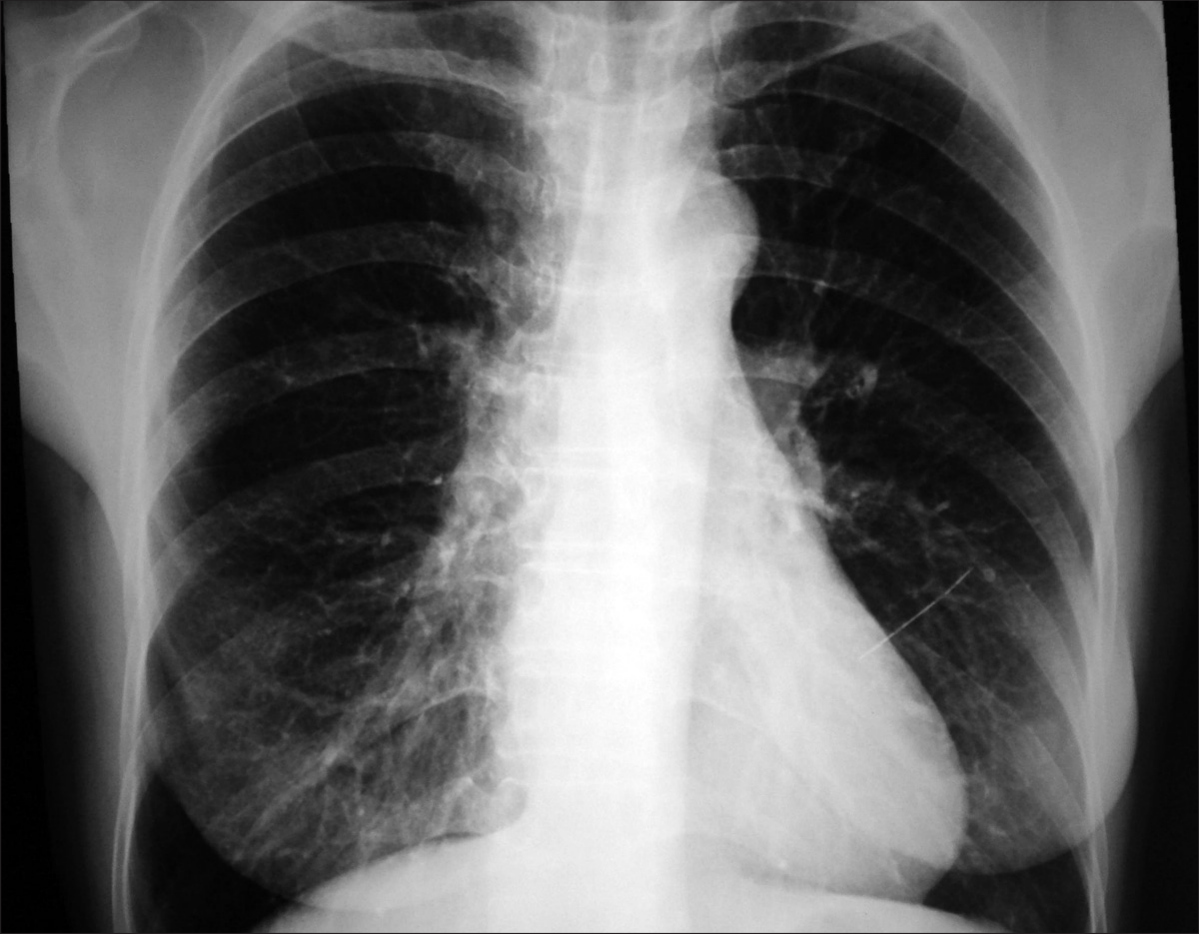
Chest X-ray showing sewing needle perpendicular to the left heart border

**Figure 2:**
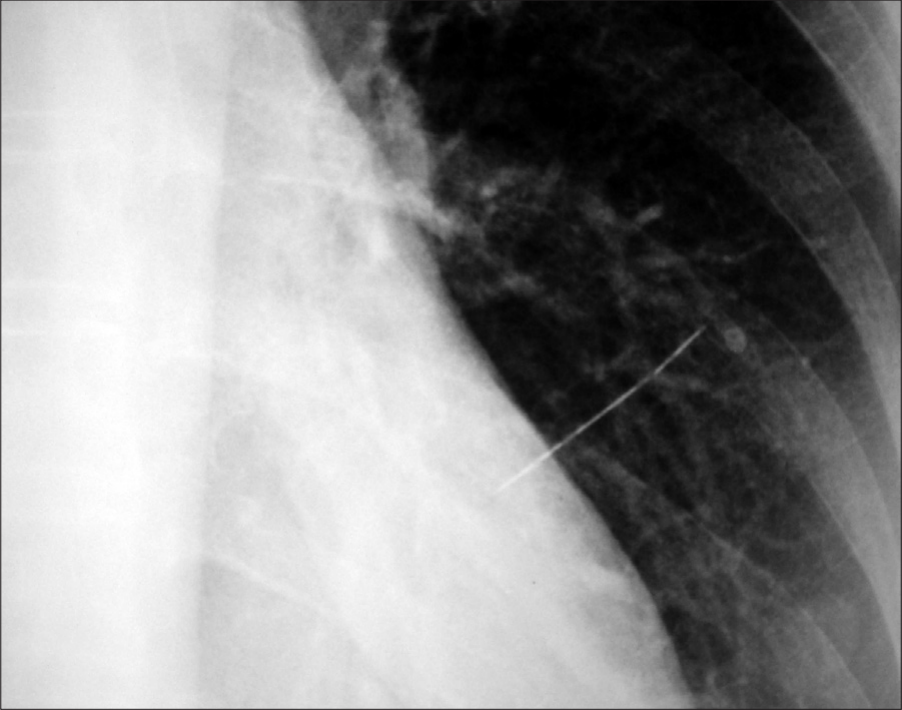
Closer view of the sewing needle with its tip well inside the left cardiac margin

Sir,

A 55-year-old female visited our outpatient clinic with history of chest discomfort for one month. Further questioning made us think that the complaint was probably non-specific. But her past medical history was surprisingly remarkable for a history of accidental aspiration of a sewing needle, 15 years back, which was then found in the left upper lobe by X-ray imaging. She was advised bronchoscopic removal of the needle at that time but she refused the procedure since she was asymptomatic. All through these years she had been asymptomatic and was actively involved in her farming work. Her vital signs and basic clinical examination was normal. A screening ECG was normal but Chest X-ray showed a linear opacity suggestive of a sewing needle perpendicular to the left cardiac border with the tip of the needle well inside the left cardiac margin [Figures 1 and 2]. We advised an urgent computerized tomography of thorax followed by brochoscopy. But she refused further investigations and treatment stating that multiple X-rays were taken on her and she has been doing well with the needle inside for 15 years! Two days later she developed chest pain of increasing intensity, which was aggravated on supine position, more so when she assumed left lateral posture. She expired during her transport to a neighboring hospital probably due to vascular injury caused by the needle.

Foreign body aspiration is very common in children, especially less than 5 years.[1] The type of foreign body depends on the environment of the child and includes peanuts, seeds, dice, whistle and plastic toys.[1] Foreign body aspiration is not uncommon among adults.[2] However, the type of foreign body among adults is different and includes bone fragments, vegetable parts, dental prosthesis, needles, metallic and plastic particle, match stick, etc.[2] Foreign body aspiration in adults can be asymptomatic[3] and occasionally present with lack of aspiration history but with complications of foreign body in the lung like pneumonia.[4]

Metallic foreign bodies (as in this case) do not produce significant symptoms since they are relatively inert and do not trigger any inflammatory changes in the lung.[5] On the contrary, foreign bodies, of organic and chemical nature, produce inflammatory changes in the lung and are often symptomatic.[5] Needles in the lung can be complicated by lung abscess and pneumothorax. This case reiterates the fact that foreign body in the lung should always be removed given the adverse consequences it can produce.
